# Suboptimal Baseline Serum Vitamin B12 Is Associated With Cognitive Decline in People With Alzheimer’s Disease Undergoing Cholinesterase Inhibitor Treatment

**DOI:** 10.3389/fneur.2018.00325

**Published:** 2018-05-09

**Authors:** Hsiao Shan Cho, Li Kai Huang, Yao Tung Lee, Lung Chan, Chien Tai Hong

**Affiliations:** ^1^Department of Neurology, Shuang Ho Hospital, Taipei Medical University, New Taipei City, Taiwan; ^2^Graduate Institute of Humanities in Medicine, Taipei Medical University, Taipei, Taiwan; ^3^Department of Psychiatry, Shuang Ho Hospital, Taipei Medical University, New Taipei City, Taiwan; ^4^Department of Psychiatry, School of Medicine, College of Medicine, Taipei Medical University, Taipei, Taiwan; ^5^Department of Neurology, School of Medicine, College of Medicine, Taipei Medical University, Taipei, Taiwan

**Keywords:** cholinesterase inhibitors, Alzheimer’s disease, vitamin B12, cognition, Mini–Mental State Status Examination, Cognitive Abilities Screening Instrument

## Abstract

**Objectives:**

Cholinesterase inhibitors (ChEIs) are the mainstream treatment for delaying cognitive decline in Alzheimer’s disease (AD). Low vitamin B12 is associated with cognitive dysfunction, and its supplementation has been applied as the treatment for certain types of reversible dementia. The present study hypothesized that baseline serum vitamin B12 is associated with the deterioration of cognitive function in people with AD undergoing ChEI treatment.

**Materials and methods:**

Between 2009 and 2016, medical records from 165 Taiwanese with mild to moderate AD who underwent ChEI treatment for at least 2 years were reviewed. Their baseline serum vitamin B12 levels were measured before treatment initiation. Their cognitive function was assessed using the Mini–Mental State Examination (MMSE) and Cognitive Abilities Screening Instrument (CASI). Student’s *t* test and multivariable logistic regression were used to analyze the association between cognitive decline and vitamin B12 level. Statistical analyses were performed using SPSS 19.0.

**Results:**

Overall, 122 participants were women. Their median age was 76 years (ranging from 54 to 91). For people with optimal baseline vitamin B12 (above the median level of 436 ng/L), the rates of MMSE and CASI decline were 0.78 ± 1.28 and 2.84 ± 4.21 per year, respectively, which were significantly slower than those with suboptimal vitamin B12 (1.42 ± 1.67 and 4.94 ± 5.88 per year; *p* = 0.007 and 0.009, respectively). After adjustment for age, sex, education level, hypertension, diabetes, history of stroke, and baseline cognitive function, the baseline serum vitamin B12 level was negatively associated with MMSE and CASI decline.

**Conclusion:**

Suboptimal baseline serum vitamin B12 level is associated with cognitive decline in people with AD undergoing ChEI treatment.

## Introduction

Alzheimer’s disease (AD) is the most common cause of dementia. Estimates have revealed that in 2050, the number of people with AD aged 65 years and older will be triple (1). Presently, no curative treatment is available for AD. The most effective symptomatic pharmacological treatment for AD is cholinesterase inhibitors (ChEIs), which delay the progress of cognitive dysfunction (2). However, the response to ChEI treatment is variable. Higher cognitive profile, a previous intellectual occupation, healthier lifestyles, being married and not living alone, a higher degree of autonomy, and lower degree of brain atrophy at baseline were associated with better response to ChEI (3). The genotype of apolipoprotein E (ApoE), a cholesterol-transporting enzyme is strongly associated with AD. However, whether ApoE genotype affects the response to ChEI in people with AD is controversial. It had been demonstrated that ApoE ε4 allele carriers were poor responders to tacrine (4); nevertheless, other studies revealed that this detrimental genotype carriers exhibited better response to donepezil (5, 6). Evidence from several prospective, randomized, placebo-controlled trials with large samples were consistent with that ApoE was not a good predictor of response to ChEIs (7–10).

Vitamin B12 has been identified to be associated with cognitive function. Vitamin B12 deficiency because of a vegetarian diet, gastric surgery, malnutrition, or alcoholism results in cognitive dysfunction, and vitamin B12 supplementation can be applied as a rescue treatment for “reversible dementia” (11). A low vitamin B12 level is associated with increasing risks of AD and behavioral and psychological symptoms of dementia in people with AD (12, 13). Although a previous study reported that vitamin B12 supplementation failed to demonstrate neuroprotection in AD (14), the significance of vitamin B12 in AD should not be underestimated.

According to the Taiwan National Health Insurance (NHI) guideline, the insurance-coverage of ChEI treatment is only available to people with AD after vitamin B12 deficiency has been ruled out. Therefore, baseline serum vitamin B12 level measurements were available for all people with approval for the ChEI prescription. The present study investigated whether the baseline vitamin B12 level was associated with the deterioration of cognitive function in Taiwanese people with AD undergoing ChEI treatment.

## Materials and Methods

### Patient Selection

This retrospective study was approved by the Joint Institutional Review Board of Taipei Medical University (TMU-JIRB) (Approval No. N201707049) and waived of informed consent was agreed by TMU-JIRB due to (1) the risk of participants is minimal and (2) waiving of informed consent is no harmful for the right of participants. Medical records from Shuang Ho Hospital between August 2009 and December 2016 were reviewed. During this period, there were 165 people with mild to moderate AD [baseline Mini–Mental State Examination (MMSE) between 10 and 26] who (1) underwent NHI-approved ChEI treatment (2) regular follow-up through cognitive function tests for at least two years. To avail of the insurance-coverage of ChEI treatment, the Taiwan NHI requires that patients should fulfill the following criteria: 1. AD diagnosis (DSM-IV diagnostic criteria of AD) and without any comorbidity affecting cognitive function, such as obvious vascular insults, vitamin B12 (≥206 ng/L), or folate deficiency, or metabolic disorder; 2. a review process to assess all medical records of the patient. The review is conducted by an NHI committee consisting of either neurologists or psychiatrists. After receiving NHI approval for ChEI treatment, patients are required to receive at least annual follow-up through the MMSE, and ChEI treatment would be discontinued for patients with MMSE decline of more than 2 points year-by-year.

The present study collected baseline medical and personal records, including age; sex; education years; medical history of hypertension, diabetes, and cerebrovascular accident (CVA); and serum vitamin B12 and folate. Since vitamin B12 level is known to be affected by several drugs (colchicine, proton pump inhibitors, histamine H2-receptor antagonists, metformin, and antiepileptic agents) and medical conditions (alcohol consumption, peptic ulcer, or gastric operation), specific drug and medical history were also recorded. All patients received MMSE and Cognitive Abilities Screening Instrument (CASI) tests at least three times: at the baseline, first follow-up, and second follow-up, with a 1-year interval. The longest follow-up period was 8 years. For ChEI treatment, donepezil (5 and 10 mg) and rivastigmine (1.5 and 4.5-mg pill and 10-mg patch) were available in Shuang Ho Hospital. People with AD were allowed to switch medications if side effects were intolerable. The deterioration of MMSE and CASI was determined by calculating the ratio of the difference in scores between the baseline and last cognitive tests (unit: score) to the time interval between two tests (unit: year).

### Statistical Analyses

All statistical analyses were performed using SPSS for Windows 10 (version 19; SPSS Inc., Chicago, IL, USA). Continuous variables are presented as mean ± SD, and categorical variables are presented as percentages with corresponding 95% confidence intervals. The differences were analyzed using Student’s *t* test. The multivariate logistic regression model was adjusted for the following variables: age; sex; education level; initial cognitive function test (either the MMSE or CASI); history of hypertension, diabetes, and CVA; and baseline folate level. A *p*-value of <0.05 was considered statistically significant.

## Results

All 165 people with AD underwent ChEI treatment for at least 2 years and were followed through annual cognitive examination (the MMSE and CASI). Their median age was 76 years (ranging from 54 to 91) while initiating treatment; 112 of them were women; and their median education years was 6 years (ranging from 0 to 16). The median level of vitamin B12 was 436 ng/L (ranging from 206 to 5,454). The distribution of serum vitamin B12 was a two-tailed normal distribution pattern (Figure S1 in Supplementary Material).

Furthermore, people with AD were categorized into two groups based on their baseline vitamin B12 levels: the group with optimal baseline vitamin B12 level (above median, 436 ng/L, *n* = 82) and suboptimal baseline vitamin B12 level (*n* = 83) (Table 1). Comparison between these two groups revealed no significant difference in most of the background demographic characteristics, including age, education years, major medical history, and baseline MMSE and CASI scores. However, more women (65 versus 47, *p* = 0.003) were in the optimal vitamin B12 group. Moreover, patients in the optimal B12 group had a significantly higher folate level. In both groups, donepezil was the main choice of ChEI treatment and the dosage of donepezil among the majority of them were titrated up to 10 mg daily. Only few of participants in both groups were exposure to drugs which may affect vitamin B12, mainly metformin.

**Table 1:** Demographic data of all patients categorized into optimal vitamin B12 (>436 ng/L) and suboptimal (≤436 ng/L) groups.

	Optimal vitamin B12 (*n* = 82)	Suboptimal vitamin B12 (*n* = 83)	*p*-Value
Women (%)	65 (79.3)	47 (56.6)	0.003
Age (years)	76.01 ± 6.93	75.41 ± 8.46	0.618
Education (years)	5.46 ± 4.81	7.10 ± 4.23	0.019
Hypertension (%)	30 (36.6)	38 (45.8)	0.269
Diabetes (%)	17 (20.7)	25 (30.1)	0.211
CVA (%)	9 (11.0)	9 (10.8)	1.000
Folate (ng/mL)	15.79 ± 10.52	11.14 ± 10.46	0.005
Baseline MMSE	18.33 ± 5.00	19.28 ± 4.38	0.197
Baseline CASI	60.30 ± 16.57	64.83 ± 13.67	0.057
ChEIs	Donepezil 5 mg, *n* = 12 Donepezil 10 mg, *n* = 50 Rivastigmine, *n* = 20	Donepezil 5 mg, *n* = 21 Donepezil 10 mg, *n* = 43 Rivastigmine, *n* = 19	0.253
Medications affecting Vitamin B12	Metformin, *n* = 7 PPI, *n* = 4 AED, *n* = 1 H2 blockers, *n* = 1	Metformin, *n* = 11 AED, *n* = 3 Colchicine, *n* = 1	
MMSE decline/year	0.78 ± 1.28	1.42 ± 1.67	0.007
CASI decline/year	2.84 ± 4.21	4.95 ± 5.88	0.009

*CVA, cerebrovascular accident; MMSE, Mini–Mental State Examination; CASI, Cognitive Abilities Screening Instrument; ChEI, cholinesterase inhibitor; PPI, proton pump inhibitor; AED, antiepileptic drug; H2, histamine H2 receptors*.

**Figure 1:**
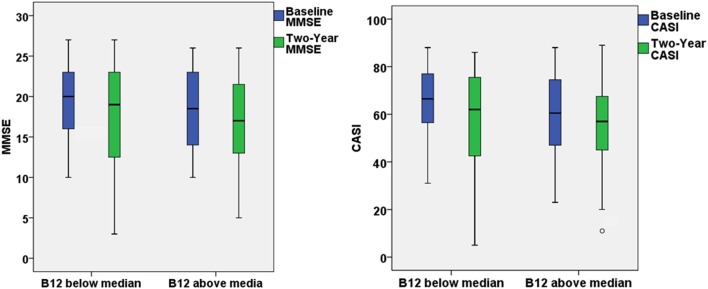
The box plots demonstrated the results of Mini–Mental State Examination (MMSE) and Cognitive Abilities Screening Instrument (CASI) at baseline and 2-year follow-up for patients with optimal (>436 ng/L) or suboptimal baseline vitamin B12 level.

The present study aimed to investigate the effect of baseline vitamin B12 on the response to ChEI treatment for cognitive decline in people with AD. After the first 2-year treatment, participants in both groups demonstrated a decline in the MMSE and CASI with highly variable results of cognitive function test within the group (Figure 1). However, if extending the follow-up period to the end of ChEI treatment (maximum follow-up period: 8 years), for patients with optimal vitamin B12 level, the corresponding cognitive decline was significantly slower than suboptimal vitamin B12 group of patients (MMSE decline: 0.78 ± 1.28 versus 1.42 ± 1.67 point/year, *p* = 0.007; CASI decline: 2.84 ± 4.21 versus 4.95 ± 5.88 point/year, *p* = 0.009) (Table 1).

If further categorized all the patients into four groups by first, second, and third quartile, the results demonstrated a similar pattern that people with higher baseline vitamin B12 level demonstrated less cognitive decline (Table S1 in Supplementary Material).

Because several factors affect cognitive decline, including age, education years, comorbidities, and baseline cognitive function before treatment, we applied a multivariable regression model to investigate the association between the baseline serum vitamin B12 level and cognitive decline. After adjustment for the aforementioned factors, the baseline vitamin B12 level was significantly and negatively associated with cognitive decline (MMSE decline, *p* = 0.009; CASI decline, *p* = 0.014) (Table 2).

**Table 2:** Multivariable regression model of the association between high vitamin B12 level and deterioration of cognitive function.

	Deterioration of Mini–Mental State Examination (MMSE)		Deterioration of Cognitive Abilities Screening Instrument (CASI)	
		
	Normalized beta coefficient	*p*-Value	Normalized beta coefficient	*p*-Value
Vitamin B12	−0.214	0.009	−0.195	0.014

*The model was adjusted for age; sex; education level; initial cognitive function test (either MMSE or CASI); history of diabetes, and CVA; and baseline folate level*.

## Discussion

The present study showed that the baseline vitamin B12 level was negatively associated with cognitive decline in people with AD undergoing ChEI treatment. The deterioration of cognitive function, as assessed by the MMSE and CASI, was significantly slower in patients with optimal baseline vitamin B12 level. Moreover, after adjustment of age, sex, education level, comorbidity, and baseline cognitive function, the baseline vitamin B12 level was negatively associated with cognitive decline. The results demonstrate that the suboptimal baseline vitamin B12 level was associated with poorer response to ChEI treatment in people with AD.

Vitamin B12 deficiency is associated with damage to the white matter in the spinal cord and in the brain, which adversely affects neuronal function. The exact mechanism underlying this association remains unknown. Damage to myelin as a result of defective methylation of myelin basic protein (MBP) has been postulated as an underlying mechanism. MBP accounts for approximately one-third of myelin protein, and demyelination in the setting of vitamin B12 deficiency may explain many of the neurologic findings, including the association with cognitive decline (15, 16). Another hypothesis is the alteration of the concentrations of cytokines, such as tumor necrosis factor-α or epidermal growth factor (17). A low vitamin B12 status leads to *S*-adenosylmethionine deficiency, which results in defective methylation reactions in the central nervous system (18). In a longitudinal study, the serum holotranscobalamin level was found to be related to cognitive performance 7 years later, even in elderly subjects without dementia, and a higher holotranscobalamin level tended to be related to higher performance in executive functions and psychomotor speed (19). Another study conducted in participants aged 45–69 years showed that participants whose vitamin B12 level was in the highest quartile had significantly higher verbal fluency scores (20). The Framingham Heart Study that included community-dwelling individuals aged 74.8 ± 4.6 years showed that plasma vitamin B12 levels ranging from 187 to 256.8 pmol/L predicted cognitive decline (21). Cohort studies have also demonstrated the association of low vitamin B12 status with cognitive dysfunction and cognitive decline (22, 23). A longitudinal cohort study that included 97 people with AD also revealed that baseline homocysteine levels showed a concentration–response relationship with the subsequent rate of decline in cognitive tests (24). However, in clinical practice, it was found that people with cognitive impairment, which is associated with vitamin B12 deficiency, did not always improve on treatment, whereas those with obvious dementia usually showed no improvement (25). Connelly suggested that the isolated use of B vitamins and folic acid is ineffective in improving cognition in people with AD, although homocysteine levels were normal or mildly increased (26). In a systematic review of four randomized controlled trials, vitamin B supplementation was found to be effective in reducing serum homocysteine levels but not in facilitating cognitive improvement (27). In a meta-analysis of 11 randomized trials involving 22,000 participants, Clarke et al. argued that 5-year dietary supplementation with B vitamins did not have any effect on cognitive domains, global cognitive function, or cognitive aging in older people (28).

The present study has some limitations. Although the medical chart review could identify numerous useful information, it was shortage of certain valuable factors for research. For instance, the present study failed to provide the ApoE genetic information, which is the strongest genetic determinant of AD. However, as aforementioned, whether ApoE genotype affects the response of ChEI was controversial. In addition, activities of daily living and instrumental activities of daily living assessments were not routinely performed in the clinical setting, so authors were not able to provide this information. On the other hand, according to the NHI guidelines about the insurance-coverage ChEI treatment, all people with AD with approved ChEI treatment are required to receive the cognitive function test annually. However, this treatment may be discontinued in patients with cognitive decline of more than 2 points in MMSE year-by-year; thus, the follow-up of cognitive function may stop. Our patients received ChEI treatment for at least 2 years, indicating that they were not the early non-responders to ChEI treatment in the first 12 months. Patients with a longer follow-up duration had a more benign course of AD. Therefore, this population was not exactly real-world people with AD but a group of partial or good responders to ChEI treatment. In addition, the vitamin B12 level of the study patients was not followed up annually. Without follow-up, no information was available to determine the correlation between the change in vitamin B12 and cognitive function and the effect of vitamin B12 supplementation on cognitive decline.

In conclusion, the present study demonstrated that suboptimal baseline serum vitamin B12 level was associated with rapid cognitive decline in people with AD undergoing ChEI treatment. Additional studies are required to delineate whether the alteration of the vitamin B12 level during the ChEI treatment period is correlated with cognitive change and to identify the specific people with AD who may be the responders to vitamin B12 supplementation.

## Ethics Statement

This retrospective study was approved by the Joint Institutional Review Board of Taipei Medical University (TMU-JIRB) (Approval No. N201707049) and waived of informed consent was agreed by TMU-JIRB.

## Author Contributions

Conception or design of the work: YL, LC, and CH. The acquisition, analysis: HC and LH. Interpretation of data for the work: HC, LH, and YL. Drafting the work or revising it critically for important intellectual content, final approval of the version to be published, agreement to be accountable for all aspects of the work in ensuring that questions related to the accuracy or integrity of any part of the work are appropriately investigated and resolved: HC, LH, YL, LC, and CH. HC and LH: both authors contributed equally to this study.

## Conflict of Interest Statement

The authors declare that the research was conducted in the absence of any commercial or financial relationships that could be construed as a potential conflict of interest.

## References

[1] Alzheimer’s Association. 2016 Alzheimer’s disease facts and figures. Alzheimers Dement (2016) 12:459–509. 10.1016/j.jalz.2016.03.001 27570871

[2] Rabins PV Blacker D Rovner BW Rummans T Schneider LS Tariot PN American Psychiatric Association practice guideline for the treatment of patients with Alzheimer’s disease and other dementias. Second edition. Am J Psychiatry (2007) 164:5–56. 18340692

[3] Gallucci M Spagnolo P Arico M Grossi E. Predictors of response to cholinesterase inhibitors treatment of Alzheimer’s disease: date mining from the TREDEM registry. J Alzheimers Dis (2016) 50:969–79. 10.3233/JAD-150747 26836164

[4] Sjögren M Hesse C Basun H Köl G Thostrup H Kilander L Tacrine and rate of progression in Alzheimer’s disease – relation to ApoE allele genotype. J Neural Transm (Vienna) (2001) 108:451–8. 10.1007/s007020170066 11475012

[5] Petersen RC Smith GE Waring SC Ivnik RJ Tangalos EG Kokmen E. Mild cognitive impairment: clinical characterization and outcome. Arch Neurol (1999) 56:303–8. 10.1001/archneur.56.3.303 10190820

[6] Bizzarro A Marra C Acciarri A Valenza A Tiziano FD Brahe C Apolipoprotein E epsilon4 allele differentiates the clinical response to donepezil in Alzheimer’s disease. Dement Geriatr Cogn Disord (2005) 20:254–61. 10.1159/000087371 16103669

[7] Wilcock GK Lilienfeld S Gaens E. Efficacy and safety of galantamine in patients with mild to moderate Alzheimer’s disease: multicentre randomised controlled trial. Galantamine International-1 Study Group. BMJ (2000) 321:1445–9. 10.1136/bmj.321.7274.1445 11110737 PMC27547

[8] Aerssens J Raeymaekers P Lilienfeld S Geerts H Konings F Parys W. APOE genotype: no influence on galantamine treatment efficacy nor on rate of decline in Alzheimer’s disease. Dement Geriatr Cogn Disord (2001) 12:69–77. 10.1159/000051238 11173877

[9] Winblad B Engedal K Soininen H Verhey F Waldemar G Wimo A A 1-year, randomized, placebo-controlled study of donepezil in patients with mild to moderate AD. Neurology (2001) 57:489–95. 10.1212/WNL.57.3.489 11502918

[10] Farlow MR Cyrus PA Nadel A Lahiri DK Brashear A Gulanski B. Metrifonate treatment of AD: influence of APOE genotype. Neurology (1999) 53:2010–6. 10.1212/WNL.53.9.2010 10599773

[11] Health Quality Ontario. Vitamin B12 and cognitive function: an evidence-based analysis. Ont Health Technol Assess Ser (2013) 13:1–45. 24379897 PMC3874776

[12] Chan A Paskavitz J Remington R Rasmussen S Shea TB. Efficacy of a vitamin/nutriceutical formulation for early-stage Alzheimer’s disease: a 1-year, open-label pilot study with an 16-month caregiver extension. Am J Alzheimers Dis Other Demen (2008) 23:571–85. 10.1177/1533317508325093 19047474 PMC10846284

[13] Shen L Ji HF. Associations between homocysteine, folic acid, vitamin B12 and Alzheimer’s disease: insights from meta-analyses. J Alzheimers Dis (2015) 46:777–90. 10.3233/JAD-150140 25854931

[14] Sun Y Lu CJ Chien KL Chen ST Chen RC. Efficacy of multivitamin supplementation containing vitamins B6 and B12 and folic acid as adjunctive treatment with a cholinesterase inhibitor in Alzheimer’s disease: a 26-week, randomized, double-blind, placebo-controlled study in Taiwanese patients. Clin Ther (2007) 29:2204–14. 10.1016/j.clinthera.2007.10.012 18042476

[15] Scott JM Folate and vitamin B12. Proc Nutr Soc (1999) 58:441–8. 10.1017/S0029665199000580 10466189

[16] Ikram MA Vrooman HA Vernooij MW den Heijer T Hofman A Niessen WJ Brain tissue volumes in relation to cognitive function and risk of dementia. Neurobiol Aging (2010) 31:378–86. 10.1016/j.neurobiolaging.2008.04.008 18501994

[17] Scalabrino G Veber D Mutti E. Experimental and clinical evidence of the role of cytokines and growth factors in the pathogenesis of acquired cobalamin-deficient leukoneuropathy. Brain Res Rev (2008) 59:42–54. 10.1016/j.brainresrev.2008.05.001 18538413

[18] Weir DG Scott JM. Brain function in the elderly: role of vitamin B12 and folate. Br Med Bull (1999) 55:669–82. 10.1258/0007142991902547 10746355

[19] Hooshmand B Solomon A Kåreholt I Rusanen M Hänninen T Leiviskä J Associations between serum homocysteine, holotranscobalamin, folate and cognition in the elderly: a longitudinal study. J Intern Med (2012) 271:204–12. 10.1111/j.1365-2796.2011.02484.x 22077644

[20] Horvat P Gardiner J Kubinova R Pajak A Tamosiunas A Schöttker B Serum folate, vitamin B-12 and cognitive function in middle and older age: the HAPIEE study. Exp Gerontol (2016) 76:33–8. 10.1016/j.exger.2016.01.011 26808046 PMC4839985

[21] Morris MS Selhub J Jacques PF Vitamin B-12 and folate status in relation to decline in scores on the mini-mental state examination in the Framingham heart study. J Am Geriatr Soc (2012) 60:1457–64. 10.1111/j.1532-5415.2012.04076.x 22788704 PMC3419282

[22] Nie T Lu T Xie L Huang P Lu Y Jiang M. Hyperhomocysteinemia and risk of cognitive decline: a meta-analysis of prospective cohort studies. Eur Neurol (2014) 72:241–8. 10.1159/000363054 25277537

[23] O’Leary F Allman-Farinelli M Samman S Vitamin B 12 status, cognitive decline and dementia: a systematic review of prospective cohort studies. Br J Nutr (2012) 108:1948–61. 10.1017/S0007114512004175 23084026

[24] Oulhaj A Refsum H Beaumont H Williams J King E Jacoby R Homocysteine as a predictor of cognitive decline in Alzheimer’s disease. Int J Geriatr Psychiatry (2010) 25:82–90. 10.1002/gps.2303 19484711

[25] Malouf R Areosa Sastre A Vitamin B12 for cognition. Cochrane Database Syst Rev (2003):CD004326 10.1002/14651858.CD004326 12918012

[26] Connelly P High dose vitamin B supplementation does not slow cognitive decline in mild to moderate Alzheimer’s disease. Evid Based Ment Health (2009) 12:86 10.1136/ebmh.12.3.86 19633254

[27] Zhang D-M Ye J-X Mu J-S Cui X-P Efficacy of vitamin B supplementation on cognition in elderly patients with cognitive-related diseases: a systematic review and meta-analysis. J Geriatr Psychiatry Neurol (2017) 30:50–9. 10.1177/0891988716673466 28248558

[28] Clarke R Bennett D Parish S Lewington S Skeaff M Eussen SJ Effects of homocysteine lowering with B vitamins on cognitive aging: meta-analysis of 11 trials with cognitive data on 22,000 individuals. Am J Clin Nutr (2014) 100:657–66. 10.3945/ajcn.113.076349 24965307 PMC4095663

