# Supplementary figures and images for: Suboptimal Baseline Serum Vitamin B12 Is Associated With Cognitive Decline in People With Alzheimer’s Disease Undergoing Cholinesterase Inhibitor Treatment
Source: Front Neurol. 2018 May 9;9:325. doi: 10.3389/fneur.2018.00325 (PMC5954104; doi:10.3389/fneur.2018.00325)

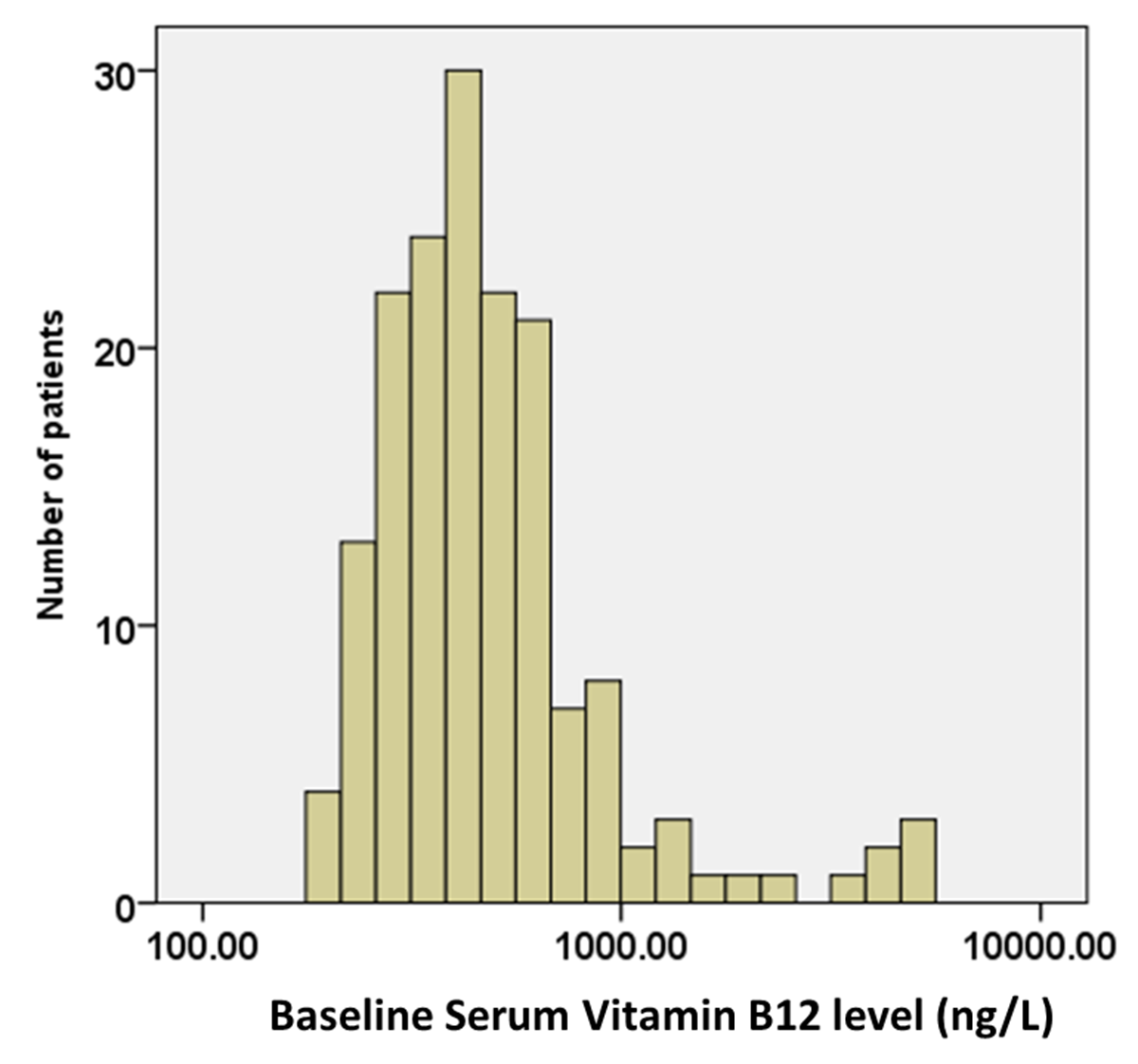

Supplement: Supplementary file 1 [file image_1.TIF]
