# Supplementary material for: Suboptimal Baseline Serum Vitamin B12 Is Associated With Cognitive Decline in People With Alzheimer’s Disease Undergoing Cholinesterase Inhibitor Treatment
Source: Front Neurol. 2018 May 9;9:325. doi: 10.3389/fneur.2018.00325 (PMC5954104; doi:10.3389/fneur.2018.00325)
Supplement: Supplementary file 2 [file table_1.DOCX]

| Baseline Vitamin B12 | Deterioration of MMSE/year | 95 % confidential interval | Deterioration of CASI/year | 95 % confidential  interval |
| --- | --- | --- | --- | --- |
| Below Q_1_ | 1.38 | 0.94-1.83 | 4.97 | 3.37-6.57 |
| Q_1_-Q_2_ | 1.47 | 0.86-2.07 | 4.94 | 2.87-7.01 |
| Q_2_-Q_3_ | 0.85 | 0.43-1.28 | 2.74 | 1.57-3.91 |
| Above Q_3_ | 0.71 | 0.32-1.09 | 2.94 | 1.44-4.44 |

Supplementary Information

Supplementary Table 1. The cognitive decline in patients divided into four groups by the first, second and third quartile.

Abbreviation: MMSE, Mini-Mental State Examination; CASI, Cognitive Abilities Screening Instrument

Figure legend

Supplementary Figure 1. The distribution of baseline vitamin B12 level among the all participants in the study.
